# The use of perineural dexamethasone and transverse abdominal plane block for postoperative analgesia in cesarean section operations under spinal anesthesia: an observational study

**DOI:** 10.1186/s12871-021-01513-4

**Published:** 2021-11-22

**Authors:** Abdisa Aga, Meron Abrar, Zewetir Ashebir, Ashenafi Seifu, Dereje Zewdu, Diriba Teshome

**Affiliations:** 1 Department of Anesthesia, Harar College of Health Science, Harar, Ethiopia; 2 Department of Anesthesia, College of Health Science, Addis Ababa University, Addis Ababa, Ethiopia; 3 Department of Anesthesia, College of Health Science, Wolkite University, PO. Box: 1362, Wolkite, Ethiopia; 4 Department of Anesthesia, College of Health Science, Debre Tabor University, Debre Tabor, Ethiopia

**Keywords:** Transverses abdominal plane block, Perineural, Dexamethasone

## Abstract

**Background:**

During transverses abdominal plane block (TAP) procedure to provide analgesia in cesarean section (CS) operation, the use of perineural dexamethasone as an additive agent may improve pain relief and may cause a prolonged block duration. This study aims to investigate whether perineural dexamethasone, when added to bupivacaine local anesthetic agent during a TAP block, may provide adequate pain relief without adverse events.

**Methods:**

This is a prospective cohort study of fifty-eight patients undergoing elective CS with spinal anesthesia. We hypothesized to perform bilateral TAP block using perineural dexamethasone as an additive agent. The patients were randomly divided into two groups using a systematic random sampling method. While one group of patients received perineural dexamethasone of 8 mg additive agent together with bupivacaine 0.25% 40 ml (Group TAPD), the other group received only bupivacaine 0.25% 40 ml in TAP block (Group TAPA). The primary outcomes are the period for the first request of postoperative pain relief medication and the numerical rating scale (NRS) pain intensity scores at 2, 6, 12, and 24 h after surgery. The secondary outcomes are comparing the 24-h tramadol and diclofenac analgesic requirements and the incidences of side effects on postoperative day one. A *p*-value of < 0.05 is statistically significant.

**Results:**

The time to first analgesic request was 8.5 h (8.39–9.79) in the TAPD group versus 5.3 h (5.23–5.59) in the TAPA group, respectively. (*p* < 0.001) The median NRS scores were significantly reduced in the TAPD group compared to the TAPA group at 6, 12, and 24 h after surgery (*p*-values < 0.001). The total analgesics consumption over 24 h postoperatively was lower in Group TAPD compared to Group TAPA (*p* < 0.05).

**Conclusion:**

An additive agent of perineural dexamethasone at a dose of 8 mg during bilateral TAP block for elective CS operation under spinal anesthesia provided better pain relief on postoperative day 1.

## Introduction

Cesarean section (CS) is commonly performed lifesaving surgical procedures to reduce fetal and maternal mortality and morbidity rates [1]. The use of regional anesthesia, including spinal or another type of peripheral block, may prevent the pain that has moderate-to-severe intensity in the first 24 h after CS operations. The postoperative moderate-to-severe pain incidence rate after CS under spinal anesthesia accounts for 77.4% of the cases. The worst pain intensity was reported at 6 h after CS operation [2, 3]. For these operations, adequate postoperative pain relief is crucial due to facilitate early ambulation, providing good infant care (including breastfeeding, maternal-infant bonding), and preventing postoperative morbidity. If acute postoperative pain after CS is inadequately treated, there is an increased incidence of chronic pain by 10–15% and some reports of post-traumatic stress syndrome. Not only these, but women with severe pain on the day after cesarean delivery will also have a 2.5 to 3-fold increased risk of postpartum depression in comparison to women with mild pain [2, 4–7]. Therefore, adequate postoperative pain management after CS is mandatory to alleviate the development of various unwanted adverse events and complications.

A regional peripheral block technique, the transversus abdominis plane (TAP) block, was first described in 2001 [8, 9]. Usually, it requires either a landmark technique or an ultrasound-guided technique for pain relief after cesarean section as part of multimodal analgesia. The landmark technique of blind “double pop” is appreciated while the needle passes the external oblique and internal oblique muscles [10–14]. The block provides blockage of subcostal nerves at the mid-axillary line before they branch anteriorly and superficially to supply the abdominal wall [15]. In several previous studies, a TAP blockade including 20 ml of 0.25% bupivacaine was administered with or without an adjuvant agent [16–18]. Various studies show the efficacy of TAP block for pain relief after CS operations. It provides adequate analgesia, decreased consumption of opioids, and reduced nausea and vomiting in the postoperative period; however, observation of better pain relief might be short-lived [19, 20].

**Fig. 1:**
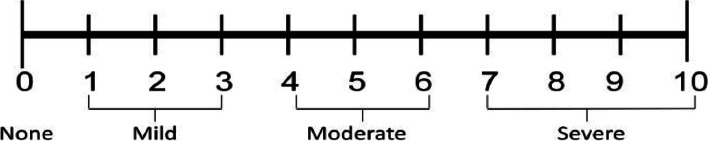
The numeric rating scale

The numeric rating scale (NRS) is a valuable pain intensity assessment tool that involves asking patients to rate their pain from 0 to 10 (11point scale) with the understanding that 0 is equal to no pain and 10 equals the worst possible pain (Fig. 1). NRS is reliable irrespective of literacy status [21]. Dexamethasone is a high-potency, long-acting glucocorticoid with minimal mineralocorticoid effect and provides relief of postoperative nausea. Its anti-inflammatory and blocking effects on neural discharge and nociception c-fibers transmission could be used as a local anesthetic adjuvant [22, 23].

The postoperative analgesic effectiveness of TAP with dexamethasone as an adjuvant is not well established. Some literature supports its efficacy [24–26], while others are against this [27, 28]. There are only nine randomized trials related to dexamethasone for TAP block during abdominal surgery in a meta-analysis [29]. A few studies suggested that dexamethasone does not affect when added to local anesthesia for pain control. Because of these controversial data and a limited number of randomized trials, we hypothesized to investigate the impact of dexamethasone additive agent in addition to bupivacaine local anesthetic agent during bilateral TAP block in CS operations under spinal anesthesia.

## Materials and methods

### Study design and patients

An institutional-based prospective cohort study was employed from January 01 to April 30, 2019, in Tikur Anbesa Hospital, a Specialized Teaching Hospital in Addis Ababa, Ethiopia. This study was performed under the Declaration of Helsinki Ethical Principles for Medical Research involving human subjects’ protocol. The study was approved by the Addis Ababa University Ethical Clearance Committee and informed written consent was secured from each study participant. Confidentiality was assured throughout the research. This study was registered at www. research registry with a registry number: *researchregistry6730*.

During a 4 months period, we included all volunteered mothers who are American Society of Anesthesiologists Physical Status (ASA-PS) classification II, undergoing elective cesarean delivery under spinal anesthesia, into this study. Although pregnant patients are usually young and healthy, the physiological changes observed during pregnancy cause various temporary changes in the human body. Therefore, according to the American Society of Anesthesiologists (ASA) description in obstetric anesthesiology guidelines, pregnancy is classified as ASA Physical Status II [30]. The exclusion criteria include; parturients with diabetes mellitus, pregnancy-induced hypertension, history of various medical illnesses, recent use of glucocorticoids, known allergy to local anesthetics, body mass index> 30 kg/m2.

### The study protocol during operation and postoperative pain management

On the arrival of the patients to the operative theatre, the anesthetist applied the essential monitors like non-invasive blood pressure, electrocardiogram, and pulse oximetry. The vital signs include; heart rate (HR), systolic blood pressure (SBP), diastolic blood pressure (DBP), peripheral arterial oxygen saturation measurement (SpO2) with pulse oximetry throughout the procedure. Spinal anesthesia is given at a sitting with 12.5 mg (2.5 ml) of 0.5% bupivacaine between L3 and L4 after observing the adequate flow of cerebrospinal fluid. A strict aseptic technique with iodine and alcohol is done for every patient before an injection. Intraoperatively, vital signs and analgesic consumption, if any, were recorded.

In our study, we hypothesized to perform bilateral TAP block using perineural dexamethasone as an additive agent. The patients were randomly divided into two groups using a systematic random sampling method. While one group of patients received perineural dexamethasone of 8 mg additive agent together with bupivacaine 0.25% 40 ml (Group TAPD), the other group received only bupivacaine 0.25% 40 ml in TAP block (Group TAPA). The TAPD group received a TAP block with 20 ml of 0.25% bupivacaine alone or with 8 mg (4 mg on each side) dexamethasone at the end of the surgery for postoperative pain relief, and the TAPA group received only bupivacaine in TAP block.

A senior anesthetist using a landmark technique performed the bilateral TAP block. Following aseptic preparation of the skin, we identified the blockade region by marking the costal margin and superior iliac spine, palpate the latissimus dorsi muscle, and forming the lumbar triangle of Petit. We used a 22G needle for injection after passing two “pop” sounds. We heard the first pop sound after we pass the external oblique muscle and the second sound heard by the internal oblique muscle. The primary outcomes were the time until the first request for postoperative analgesia and the numerical rating scale (NRS) pain intensity scores at 2, 4, 6, 12, and 24 h after surgery. The secondary outcome was the comparison of the 24 h of analgesic requirement in mg of tramadol and diclofenac for both groups and in addition the comparison of the incidences of side effects on postoperative day one.

### The sampling procedures and the sample size calculation

**Fig. 2:**
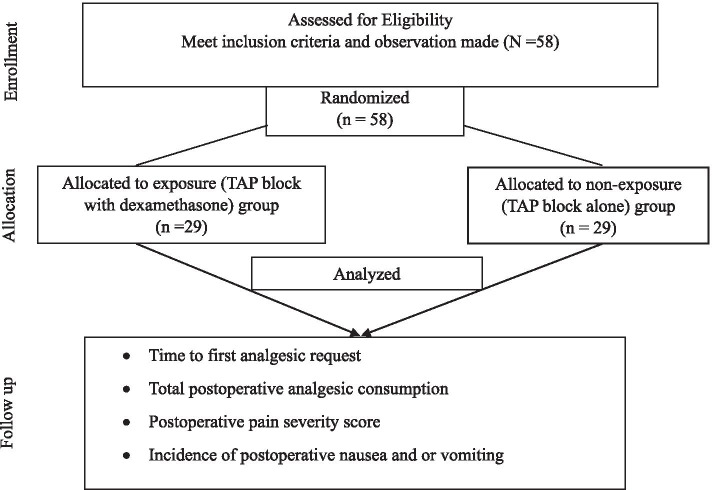
The consortium diagram of study participants

The postoperative pain management standards for cesarean delivery in a study hospital are bilateral TAP block with 40 ml of 0.25% bupivacaine with or without 8 mg dexamethasone as an adjuvant (20 ml with or without 4 mg dexamethasone as an adjuvant on each side). A situational analysis was done to estimate the number of parturients who receive either TAPD or TAPA. Accordingly, about 1192 CS annually or 298 elective CS mothers received either TAPD or TAPA (157 parturient TAPD and 141 parturient TAPA) per 4 months. A systematic random sampling technique was used to select study participants. The sampling interval k was determined to be 5 using the formula: k = N/n (298/58); *n* = total sample size, *N* = population per 4 months. Each participant had about a 20% equal probability of being included in the study. A schedule list of elective CS was used as a sampling frame, and the first random start was determined by a simple lottery method. Then the skipping interval was used for the rest of the study participants till the study ended. The selected study participants were allocated to either group based on what they had been given for postoperative pain management plan (TAPD or TAPA) (Fig. 2).

We calculated the sample size from the primary outcomes with a pilot study conducted before the actual research using the G- power, version 3.1.9.2. The main outcome measure was the time to first analgesic request because we took the largest sample size to appreciate the possible difference. The mean time to first analgesic requests in both groups of patients were 7.15 ± 2.7 h and 5.5 ± 1.32 h in the TAPD and TAPA groups, respectively. These values are used in the G power with alpha 0.05 and a capacity of 80 to calculate the sample size. Therefore, it gave us a sample size of 52. By taking a 10% attrition rate, we determined that 58 patients are required. Therefore, we enrolled 58 patients in the study.

### The collection of data

We collected our data using pretested questionnaires with multiple close-ended questions on respondents following informed consent by trained two Nurses data collectors. They are unaware of study groups. On the morning of the surgery, the data collector instructs the patients on self-reporting their pain using the eleven-point NRS score of 0 to 10 [21]. The scale consists of horizontal lines ranging from 0 (no pain) to 10 (worst imaginable pain). The chart review provided data on demographic and intraoperative variables.

The pain intensity was followed and recorded as: mild (NRS:0–3), moderate (NRS: 4–6), and severe (NRS: 7–10). The NRS score was recorded postoperatively at 2, 4, 6, 12, and 24 h. At the times of pain evaluation, the heart rate, the mean arterial blood pressure, respiratory rate, and pulse oximetry (SPO2) values were recorded.

The time between the administration of regional blockade and the first pain relief medication request was recorded from the patient chart after admission to the ward. The 24-h postoperative analgesic requirement, any incidence of adverse events were recorded.

### Statistical analysis

We entered and analysed the data using SPPS 20.0 software for Windows (SPSS Software, CA, USA). We checked the data for normality using Shapiro–Wilk test. We checked the homogeneity of variance using Levene’s test. We performed an analysis of the student t-test test for normally distributed numerical variables between study groups. We completed an analysis of the Mann-Whitney U test for non-normally distributed numerical variables (like pain severity score in NRS, time to first analgesic request, total analgesic consumption). A one-way repeated measured analysis of variance (ANOVA) was conducted to evaluate within group difference in severity of postoperative pain (NRS) when measured at 2, 4, 6, 12, and 24 h postoperatively for the both TAPA and TAPD groups. Numeric data were described as mean ± SD for symmetric and median (interquartile range) for asymmetric numeric data. We presented the categorical variables as frequency and percentage, and we used the Chi-square test and Fisher’s exact test for statistical differences between groups. A *p*-value of < 0.05 with a power of 80% is considered statistically significant.

### Operational definitions

#### Failed TAP block

The NRS score is four times more at the 2nd hour postoperatively, which provided a failed TAP block.

#### Time to first analgesia request

A time in minutes from the end of surgery to the first-time analgesia was given.

#### Total analgesic consumption

The total of analgesia medications that we administer in 24 h in the postoperative period.

#### Numeric rating scale

This is a valuable pain intensity assessment tool that involves asking patients to rate their pain from 0 to 10 (an 11point scale) to understand that 0 equals no pain and 10 equals the worst possible pain [14].

#### Postoperative nausea and vomiting

A patient experiences at least one episode of either nausea or vomiting within 24 h postoperatively. We assessed the complaint of nausea and or vomiting, and the patient was given a score according to nausea and or vomiting score described by McDonnell et al. [15]

0 - No nausea/vomiting in past 24 h.

1 - Nausea in past interval.

2 - Vomiting in past interval.

## Results

### The comparison of demographic and perioperative characteristics

During the study period, a total of 58 patients were analysed based on whether they received TAPD or TAPA at the end of surgery for postoperative analgesia. There was no statistically significant difference between the two groups concerning age, height, weight, BMI, Gravidity, ASA physical status (*P*-value > 0.05) (Table 1).

**Table 1:** Demographic and perioperative characteristics study participants

	Group TAPD *N* = 29	Group TAPA *N* = 29	*P*-value
Age (year)^a+^	28.44 ± 2.81	26.41 ± 3.407	0.211
Height (m)^a+^	1.61 ± 0.0402	1.59 ± 0.0385	0.698
Weight (kg)^a+^	71.06 ± 6.803	69.48 ± 6.21	0.40
BMI (Kg/m^2^)^a+^	25.92 ± 2.046	25.969 ± 2.461	0.831
Operation surgery (min)^a+^	48 ± 7.64	45 ± 5.85	0.131
Gravidity^b+^			
One	8	11	0.158
Two	18	13	
Three and above	3	5	

*P-value: *p* < 0.05 is statistically significant; N.B: ^a^ mean ± SD, ^b^ frequency of cases, +: the comparison between the two groups using Chi-Square Test, TAPD: transverse abdominis plane block with dexamethasone, TAPA: transverse abdominis plane block without dexamethasone

### The comparison of postoperative pain relief characteristics between the groups

The Mann-Whitney U-test showed that the median time between administration of regional blockade and the time to first analgesic request in minutes was prolonged. At the same time, postoperative analgesics consumption was reduced in the dexamethasone group significantly compared to the non-dexamethasone TAP group (Table 2). The difference is seen based on the doses and frequency in Table 2 (*p*-values of < 0.05).

**Table 2:** The comparison of analgesia related parameters between the groups

	Group TAPD *N* = 29	Group TAPA *N* = 29	*P*-value
First analgesic requirement time (minutes) #c	510 (503.58–587.58)	318 (313.51–335.48)	< 0.001*
Total analgesics consumption #c			
Tramadol in mg (IV)	50 (37.82–62.52)	100 (69.77–88.84)	0.001*
Diclofenac in mg (IM)	75 (42.31–71.47)	75 (73.91–91.60)	0.003*

*P-value: *p* < 0.05 is statistically significant, N.B: #: median (Interquartile range); #c: the comparison between two groups using Mann-Whitney U-test, *TAPD* Transverse abdominis plane with dexamethasone, *TAPA* Transverse abdominis plane without dexamethasone

### The comparison of postoperative pain intensity by a numeric pain rating scale

We performed in comparison of the two-group data for NRS scores a Mann-Whitney U-test. This statistical analysis revealed that a significant reduction in median NRS scores at 6th, 12th, and 24th hours in the TAP block with dexamethasone group (Group TAPD) as compared to TAP block without dexamethasone group (Group TAPA) at 6, 12, and 24 h after surgery (*p* < 0.001, *p* < 0.001, *p* < 0.001, respectively). But there were no significant differences in comparison between the TAPD and TAPA groups for pain intensity values represented by NRS at the 2nd and 4th hours (*p* > 0.05) (Table 3).

**Table 3:** The comparison of postoperative pain severity by numerical rating scale (NRS) score among study participants at time points of 2, 4, 6,12 and 24 h after surgery

Postoperative pain severity score (NRS)			
	Group TAPD *N* = 29	Group TAPA *N* = 29	*P*-value
2 h #c	1 (0–1)	1 (0–1)	0.84
4 h #c	2 (0–3)	2 (2–3)	0.21
6 h #c	2 (2–3)	4 (4–3)	< 0.001*
12 h #c	3 (3–4)	4 (4–5)	< 0.001*
24 h #c	3 (3–4)	4 (4–5)	< 0.001*

*P-value: *p* < 0.05 is statistically significant, N.B: #: median (Interquartile range); #c: the comparison between two groups using Mann-Whitney U-test, *TAPD* Transverse abdominis plane with dexamethasone, *TAPA* Transverse abdominis plane without dexamethasone

A one-way repeated measured analysis of variance (ANOVA) was conducted to evaluate within group difference in severity of postoperative pain (NRS) when measured at 2, 4, 6, 12, and 24 h postoperatively for the both TAPA and TAPD groups. The results of the ANOVAs indicated a significant Postoperative pain severity score, Wilks’ Lamda =0.16, F (3, 26) = 44.43, *p* < 0.01, partial Etta squared = 0.84 for TAPA group, and Wilks’ Lamda = 0.08, F (4, 25) = 44.43, *p* < 0.01, partial Etta squared = 0.92 for TAPD group. Thus, there is significant differences within groups. Follow up comparison in both groups indicated that each pairwise difference was significant, *p* < 0.01.

### Prevalence of nausea and vomiting

The prevalence of postoperative nausea and vomiting was 27.5%. The proportions of patients with nausea and vomiting were statistically significantly lower in TAP with dexamethasone group than TAP without dexamethasone group (*p* < 0.05). We did not observe any other complications in both groups.

## Discussion

In this study, all TAP blocks performed were successful, and confounding factors such as demographic characteristics, duration of surgery, gravidity, and ASA status were comparable between the groups. The difference in time to first analgesia request, pain severity, and total 24 h analgesic consumption between groups was likely due to the perineural dexamethasone in the exposure variable.

This study showed that the median time for the first analgesic request was significantly prolonged in the TAPD block group compared to the TAPA group (*p* = 0.00). Similar to our findings, studies were done by Amany AS et al. [25], Sharma UD et al. [31], Fouad HA et al. [32], Sachdeva J et al. [33], and Zemedkun A et al. [34] found that the mean duration of the time to first analgesic requests was statistically significantly longer in the TAPD group as compared to the TAPA group after abdominal surgeries and CS under spinal anesthesia (*p*-values < 0.05).

In the conflict of our finding, a study done by Huang SH et al. [28] failed to disclose a statistically significant difference in duration to first rescue analgesia. This might be due to the block is given following general anesthesia in their case. In our study, the block is given after spinal anesthesia.

In our study, there was significantly reduced postoperative pain (NRS score) in the TAPD group (*p* < 0.05) at the 6th, 12th, and 24th hours as compared to the TAPA group, and there was no significant difference at 2nd and 4th hour between two groups. This is in line with studies conducted by the Raghukumar M et al. [35], Sharma UD et al. [31], Zemedkun A et al. [34], and Deshpande J et al. [36], which showed that a statistically significant reduced postoperative pain severity score in VAS in group TAPA groups at different point of time (*p*-values < 0.05).

In contrary to our findings, studies were done by Amany AS et al. [25] found that statistically significant decrease in pain score at 2nd hours and 4th hours postoperatively in a TAPD group as compare control group (TAPA) and Wegner R et al. [27] failed to demonstrate a statistical significance between the groups concerning postoperative pain severity score. This might be because the block is given following general anesthesia in their case. In our study, the block is given after spinal anesthesia.

With regards to total postoperative analgesic consumption, the median (IQR) of 24 h total diclofenac and tramadol were significantly reduced in the TAPD group as compared to the TAPA group (*p*-values < 0.05). In agreement with our finding, studies conducted by Sachdeva J et al. [33], Deshpande J et al. [36], and Fouad HA et al. [32] reported that there is a statistically significant reduction of 24-h total analgesic consumption in the TAPD group as compared to TAPA.

In conflict with our finding, a study done by Huang SH et al. [28] failed to disclose a statistically significant difference in postoperative total analgesic consumption. This might be due to the block is given following general anesthesia in their case; in our study, the block is given after spinal anesthesia.

The prevalence of postoperative nausea and vomiting in a current study is statistically significantly lower in the TAPD group compared to the TAPA (*p* = 0.04). This finding was also in line with the survey done by Sachdeva J et al. [33] and Amany AS et al. [25], as the prevalence of PONV is reported statistically significantly lower in the TAPD compared to the TAPA group.

### Limitations of the study

This study is limited to a single-center, and it is not a randomized controlled study. The sample size has been provided and was acceptable to detect differences in pain intensity between the two groups of patients. However, the study needs to be conducted on a larger group of patients.

### Strength of the study

Study participants were homogeneous.

## Conclusion

This study aimed to investigate whether perineural dexamethasone, when added to bupivacaine local anesthetic agent in TAP block, may provide adequate pain relief and no adverse events in elective CS surgeries under spinal anesthesia. The primary outcomes are the time until the first request for postoperative analgesia and the numerical rating scale (NRS) pain intensity scores at 2, 12, and 24 h after surgery. The secondary outcomes are tramadol and diclofenac analgesic consumption for 24 h in mg and incidences of side effects on postoperative day one. Finally, we showed that bilateral TAP block providing perineural dexamethasone of 8 mg as an additive agent to bupivacaine prolonged time to first analgesia request, decreased analgesic consumption, and provided better pain relief during the first 24 h postoperatively.

## Data Availability

The datasets used and/or analysed during the current study are available from the corresponding author on reasonable request.

## Abbreviations

AAU: Addis Ababa University
ASA: American Society of Anesthesiologists
CS: CS
NRS: Numeric Rating Score
TAP: Transverse Abdominis Plane block TAPD: Transverse Abdominis Plane block with Dexamethasone
TAPA: Transverse Abdominis Plane block without dexamethasone
